# Biological Mechanisms of Enterotoxigenic *Bacteroides fragilis* Toxin: Linking Inflammation, Colorectal Cancer, and Clinical Implications

**DOI:** 10.3390/toxins17060305

**Published:** 2025-06-16

**Authors:** Seyedesomaye Jasemi, Paola Molicotti, Milena Fais, Ilaria Cossu, Elena Rita Simula, Leonardo A. Sechi

**Affiliations:** 1 Department of Biomedical Sciences, Division of Microbiology and Virology, University of Sassari, 07100 Sassari, Italy; molicott@uniss.it (P.M.); faismilena@gmail.com (M.F.); i.cossu2@studenti.uniss.it (I.C.); ersimula@uniss.it (E.R.S.); 2 Struttura Complessa Microbiologia e Virologia, Azienda Ospedaliera Universitaria Sassari, 07100 Sassari, Italy

**Keywords:** enterotoxigenic *Bacteroides fragilis* (ETBF), *Bacteroides fragilis* toxin (BFT), non-toxigenic *Bacteroides fragilis* (NTBF), colorectal cancer (CRC), polymerase chain reaction (PCR)

## Abstract

Enterotoxigenic *Bacteroides fragilis* (ETBF) has emerged as a gut microbiome pathogen that can promote intestinal inflammation and contribute to colorectal cancer (CRC). Its principal virulence factor, the *Bacteroides fragilis* toxin (BFT), is a zinc-dependent metalloprotease that disrupts epithelial barrier integrity, initiates inflammatory signaling pathways, and enhances epithelial proliferation. Although growing evidence supports a link between ETBF and CRC, some inconsistencies across studies highlight the need for further investigation into the molecular mechanisms underpinning BFT-mediated pathogenesis. This review examines the biological structure and activity of BFT, with a focus on its role in epithelial injury, inflammatory responses, and tumorigenesis. In addition, we discuss current challenges in the detection and characterization of ETBF and BFT, including technical limitations in clinical diagnostics and methodological variability across studies. Recent advances in multi-omics technologies, molecular diagnostics, nanobody-based detection platforms, and probiotic intervention are also highlighted as promising avenues for improving ETBF identification and therapeutic targeting. Future research integrating systematic molecular profiling with clinical data is essential to enhance diagnostic accuracy, elucidate pathophysiological mechanisms, and develop effective interventions against ETBF-associated diseases.

## 1. Introduction

The human gastrointestinal (GI) tract harbors a vast and diverse population of microorganisms, known as the intestinal microbiota, with an estimated density of 10^11^ bacteria per gram of feces [1]. This complex ecosystem plays a crucial role in maintaining human health by regulating immune responses, supporting metabolic processes, and providing protection against pathogenic organisms [2,3]. Disruptions in the composition or function of the microbiota, a condition referred to as dysbiosis, have been closely associated with a broad spectrum of diseases, including cardiovascular, neurological, gastrointestinal, and inflammatory disorders [4,5].

The dominant intestinal microbiota consists of two major phyla, *Bacteroidetes* and *Firmicutes* [6], with species from the genus Bacteroides accounting for approximately 25–50% of the microbial population [7]. Although *Bacteroides fragilis* represents only about 0.5–1% of the cultured fecal bacteria [8], it has attracted considerable attention due to its dual role in host interactions, exhibiting both beneficial and pathogenic characteristics [9,10,11,12]. *Bacteroides fragilis* exists in two main variants: non-toxigenic *Bacteroides fragilis* (NTBF), which does not produce toxin, and enterotoxigenic *Bacteroides fragilis* (ETBF), which produces *Bacteroides fragilis* toxin (BFT) [13,14]. The key difference between these two forms is the ability of ETBF to produce BFT, a feature that transforms a potentially beneficial commensal into a pathogenic organism [8,9], which has been proposed as a potential probiotic due to its anti-inflammatory properties, which are primarily mediated through the stimulation of regulatory T (Treg) cells, suppression of Th2 immune responses, and enhancement of IL-10 secretion, largely driven by the production of capsular components such as polysaccharide A (PSA). Additionally, NTBF contributes to host defense by inhibiting the colonization of pathogens, including *Clostridioides difficile*, through the activation of Paneth cells and the induction of antimicrobial peptide secretion [15]. In an experimental study, the preventive effects of the *B. fragilis* ZY-312 strain was evaluated in a murine model of *C. difficile* infection (CDI). Mice challenged with *C. difficile* strain VPI 10463 developed severe diarrhea, disruption of intestinal barrier function, and high mortality rates. In contrast, mice pretreated with *B. fragilis* ZY-312 showed significantly improved survival, preservation of intestinal barrier integrity, evidenced by increased expression of ZO-1 and MUC-2, and enhanced microbial diversity within the gut, thereby preventing pathogen colonization [16].

**Figure 1:**
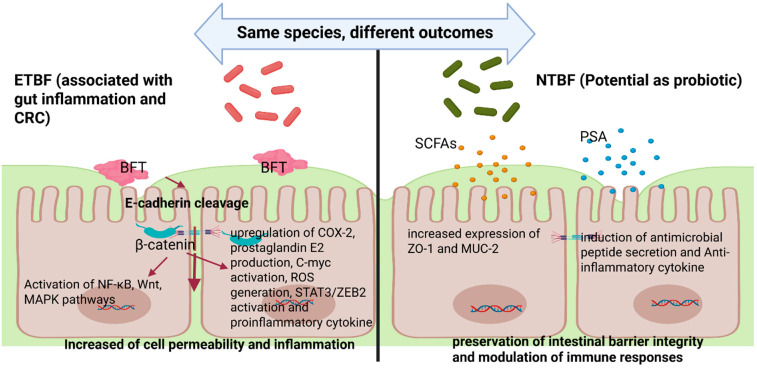
Differential effects of enterotoxigenic *Bacteroides fragilis* (ETBF) and non-toxigenic *Bacteroides fragilis* (NTBF) on intestinal epithelial cells. ETBF produces *Bacteroides fragilis* toxin (BFT), leading to E-cadherin cleavage, activation of β-catenin, upregulation of inflammatory pathways (NF-κB, Wnt, MAPK), COX-2, prostaglandin E2 production, C-myc and STAT3/ZEB2 activation, ROS generation, and proinflammatory cytokine release, ultimately resulting in increased intestinal permeability and inflammation. In contrast, NTBF promotes gut homeostasis through the production of short-chain fatty acids (SCFAs) and polysaccharide A (PSA), upregulation of barrier-protective molecules (ZO-1, MUC-2), induction of antimicrobial peptides, and secretion of anti-inflammatory cytokine.

These contrasting outcomes of ETBF and NTBF on epithelial integrity and immune modulation are summarized in Figure 1.

Furthermore, a study conducted by Chan et al. demonstrated that NTBF can reduce bacteria-driven chronic colitis and tumor development independently of polysaccharide A (PSA) production [17]. In a murine model of antibiotic-associated diarrhea (AAD), the NTBF strain ZY-312 was shown to alleviate diarrhea symptoms, restore intestinal barrier integrity, and promote the reconstruction of the gut microbiota [10]. In addition, in a dextran sulfate sodium (DSS)-induced colitis mouse model, NTBF was shown to promote colonic mucosal regeneration by stimulating interleukin-22 (IL-22) production. This, in turn, activates the STAT3 signaling pathway, thereby enhancing epithelial cell proliferation and strengthening the integrity of the intestinal barrier [18].

In contrast, substantial evidence from epidemiological, molecular, animal model, and clinical studies has underscored the pathogenic role of ETBF strains in the development of gastrointestinal inflammatory conditions, including diarrhea [19,20,21], colitis [19,22], and colorectal cancer [23,24,25]. Additionally, asymptomatic colonization with ETBF (ranging from 6.2% to 20%) appears to be relatively common among adults, complicating its epidemiological characterization and raising concerns about its potential for silent pathogenicity [26,27,28,29].

Despite the existence of previous reviews—such as the systematic review by Scott et al. (2022) that evaluated the role of ETBF in colorectal cancer with a focus on epidemiological heterogeneity and detection challenges, and the mechanistic analysis by Cheng et al. (2020) that explored BFT-induced oncogenic pathways—there remains a lack of a comprehensive and integrative synthesis [30,31]. This review aims to bring together recent findings on the genetic and protein structures of BFT, highlight its roles in chronic inflammation and tumor development, and discuss key challenges in the identification and characterization of ETBF strains. Moreover, emerging multi-omics approaches, and early detection strategies are reviewed to provide insights that may guide future research directions in this field.

## 2. Molecular and Genetic Structure of *Bacteroides fragilis* Toxin (BFT)

BFT is the main pathogenicity factor of ETBF. The *bft* gene encoding this toxin is located within a pathogenicity island (BfPAI) of approximately 6 kilobases, which is typically inserted into a 65-kilobase conjugative transposon (CTn86) on the bacterial chromosome [32]. This island contains both the *bft* and *mpII* (another metalloprotease) genes and is characteristically present in ETBF strains [33]. NTBF strains lack the BfPAI; however, some NTBF isolates harbor the flanking regions of CTn86, enabling potential horizontal transfer and acquisition of the pathogenicity island from ETBF strains. Based on chromosomal structure and toxin gene presence, *Bacteroides fragilis* strains are classified into three patterns as follows: Pattern I: ETBF strains containing both the flanking region and the *bft* gene; Pattern II: NTBF strains with an intact chromosome, lacking both the flanking region and the *bft* gene; and Pattern III: NTBF strains harboring the flanking region without the pathogenicity island or the *bft* gene [32,33].

The *bft* gene consists of 1191 nucleotides with a GC content of approximately 39%. Three major alleles have been identified—*bft-1*, *bft-2*, and *bft-3*, encoding the BFT-1, BFT-2, and BFT-3 isoforms, respectively. Moreover, a 700-base-pair upstream regulatory region is critical for the optimal production of BFT in ETBF [14,34,35]. The *bft* gene comprises three isotypic variants, among which *bft-2* exhibits the highest pathogenic potential, followed by *bft-1* and *bft-3* [36,37]. Notably, the simultaneous presence of multiple isoforms within a single strain has not been reported; nonetheless, some strains harbor two homologous copies of a specific isoform. Based on previous global epidemiological studies, including those from Iran, Turkey, and the United States, *bft-1* has been identified as the most prevalent isotype of the BFT, whereas BFT-3 has been predominantly identified in East Asian regions, including Iran, Japan, and Vietnam [26,34,37].

**Figure 2:**
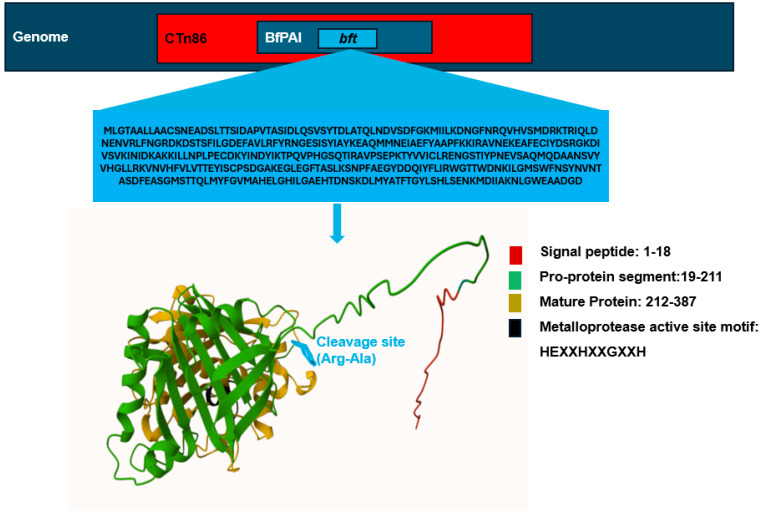
Genomic context and structural domains of *Bacteroides fragilis* toxin (BFT). The *bft* gene is located within the ~6 kb *Bacteroides fragilis* pathogenicity island (BfPAI), typically integrated into the 65 kb conjugative transposon CTn86 on the bacterial chromosome. The schematic illustrates the genomic organization of CTn86, BfPAI, and the *bft* gene flanked by regulatory and accessory elements. The amino acid sequence of the BFT precursor is shown below, with functional domains color coded as follows: signal peptide (residues 1–18, red), pro-domain (residues 19–211, green), and mature toxin (residues 212–387, yellow). The zinc-dependent metalloprotease catalytic motif (HEXXHXXGXXH) is highlighted within the mature domain (black).

The BFT holotoxin is expressed as a 397-amino-acid protein with an approximate molecular mass of 44.5 kilodaltons [14]. Structurally, the holotoxin is organized into three functional domains: the signal peptide region, the proprotein segment, and the mature toxin [14,35]. Figure 2 provides an overview of the genomic organization of the *bft* gene, along with the domain architecture and three-dimensional structure of the BFT holotoxin.

The signal peptide domain, composed of 18 amino acids, is essential for directing the holotoxin to the bacterial membrane. Following secretion, the holotoxin exists in a biologically inactive zymogen form (proBFT), in which the ~170-amino-acid pro-domain masks the active site, preventing enzymatic activity.

Proteolytic cleavage at the Arg211–Ala212 junction is required to remove the inhibitory pro-domain and release the mature active toxin [38,39,40]. The 190-amino-acid catalytic domain (CD) of mature BFT contains a characteristic zinc-dependent metalloprotease motif (HEXXHXXGXXH), which is responsible for its enzymatic activity, specifically the degradation of key proteins such as E-cadherin, actin, gelatin, and casein [38,41,42]. This motif, alongside a downstream methionine residue (Met366) embedded in a tight 1,4-β-turn known as the Met-turn, classifies BFT within the metzincin clan of metalloproteases [38,39,43]. The motif includes three histidines that coordinate the catalytic zinc ion and a glutamate residue that functions as a general acid/base during catalysis. Site-directed mutagenesis of critical residues within this core—such as His348, Glu349, His352, Gly355, His358, and Met366—results in a dramatic reduction or complete loss of enzymatic activity, impairing the toxin’s ability to induce morphological alterations in epithelial cells [44]. Intriguingly, its catalytic domain shares high structural similarity with mammalian adamalysins/ADAMs, suggesting a rare horizontal gene transfer event from eukaryotes to *B. fragililis* [45]. Interestingly, although BFT shares this catalytic architecture with other metzincins, it lacks upstream sequence homology, suggesting that it may represent a unique prototype within the clan [39]. Furthermore, structural integrity of the C-terminal region appears to be essential, as the deletion of even two residues significantly diminishes enzymatic function, while the removal of eight or more leads to complete functional inactivation, despite normal secretion and processing [46]. These findings collectively underscore the critical role of both the zinc-binding motif and the C-terminal architecture in maintaining the proteolytic activity of BFT.

In the BFT-2 isoform, an amphipathic region located at the carboxyl terminus is proposed to facilitate toxin penetration into the host cell membrane and contribute to the formation of ion channels. BFT-1 and BFT-2 exhibit resistance to trypsin and maintain stability across a broad pH range [36,47].

Regulation of BFT expression is mediated, at least in part, by the two-component regulatory system RprXY, which functions as a negative regulator. Overexpression of RprXY has been shown to markedly suppress *bft* gene expression and reduce BFT production, as demonstrated in experimental models, including Muc2-deficient mice [48]. Recent evidence has revealed that BFT is not freely secreted into the extracellular environment but is packaged with outer membrane vesicles (OMVs). This vesicle-mediated delivery plays a pivotal role in facilitating epithelial disruption and may represent a key mechanism in the pathogenesis of intestinal inflammation [49].

## 3. ETBF-Associated Diseases and Underlying Mechanisms

Until the 1970s, *Bacteroides fragilis* was primarily recognized as an opportunistic symbiont within the GI microbiota [50,51]. Notably, *Bacteroides fragilis* remains the most frequently isolated anaerobic bacterium from human clinical specimens associated with anaerobic infections [52,53]. Although it comprises only approximately 0.5–1% of the normal GI microbiota, translocation to extraintestinal sites can lead to a range of severe infections, including intra-abdominal sepsis, genital tract infections, subcutaneous abscesses, endocarditis, pericarditis, and bacteremia [53,54,55]. In particular, *Bacteroides fragilis*-associated bacteremia carries a significant mortality rate of approximately 19% [55].

Since the early 1980s, evidence has emerged suggesting that certain strains of *Bacteroides fragilis* may act beyond the role of an opportunistic pathogen. One of the first pivotal studies in this field was conducted by Myers and colleagues, who isolated *Bacteroides fragilis* strains with enterotoxin-like activity (BFEL) from the feces of 24- to 48-h-old lambs with diarrheal disease in the Northern Rocky Mountain region of the United States [56]. Pure cultures of *Bacteroides* obtained from diarrheal lambs induced significant fluid accumulation in lamb and calf intestinal loop models [56]. Three distinct BFEL serogroups were identified among the diarrheal cases. Furthermore, oral inoculation of newborn colostrum-fed lambs with viable BFEL strains resulted in diarrhea, lethargy, and, in one case, death within 32 h post-inoculation, closely resembling the clinical signs observed in natural infections. Subsequent studies successfully isolated ETBF strains from fecal samples of other farm animals, including lambs, calves, and pigs [56,57,58]. In addition, in a follow-up study by Meyer and colleagues, ETBF was isolated from the fecal specimens of 8 out of 44 individuals with diarrhea. In rabbit models, ETBF strains induced mucous and hemorrhagic diarrhea as well as moderate to severe necrotizing colitis, whereas non-toxigenic strains failed to produce such pathological changes. Collectively, these findings underscore the significant role of ETBF in promoting intestinal damage, including diarrhea and colitis [59].

In 2006, Toprak et al. demonstrated for the first time the link between ETBF and colorectal cancer (CRC) [60]. In their study, stool samples from 73 patients with CRC and 59 control individuals were evaluated. After the isolation of *Bacteroides fragilis* and its identification by conventional methods, the presence of the *bft* gene was examined using polymerase chain reaction (PCR). The results showed that 38% of isolates from CRC patients carried the *bft* gene, while this figure in the control group was only 12% (*p* = 0.009) [60]. Since the 1980s, numerous studies have demonstrated that the frequency of ETBF strains in biopsy, stool, and mucosal samples from patients with diarrhea, inflammatory bowel disease, and colorectal cancer is significantly higher than in healthy individuals [60,61,62,63,64]. These findings suggest a potential role for ETBF in the initiation and progression of intestinal inflammatory disorder. Nevertheless, some studies have reported no clear association between *Bacteroides fragilis* and disease [65,66]. These inconsistencies may be due to variations in study populations, methodologies, or the failure to distinguish between toxigenic and non-toxigenic strains.

The pathogenesis of BFT, a zinc-dependent metalloprotease, begins with its highly specific binding to a still-unidentified membrane receptor on colonic epithelial cells (CECs). This interaction is strictly dependent on physiological temperature (37 °C) and requires the proteolytically active form of the toxin; catalytically inactive mutants are unable to bind [38,67]. Binding occurs in a polarized fashion—at the apical membrane in differentiated HT29/C1 cells and basolateral membrane in T84 crypt-like cells—suggesting that the receptor redistributes during epithelial differentiation [67]. These features underscore the specificity of BFT–receptor interactions, which serve not only as the initiating step of the pathogenic cascade but also as a trigger for intracellular signaling pathways that compromise epithelial integrity

One of the earliest outcomes of BFT-receptor engagement is the cleavage of E-cadherin, a key cell–cell adhesion molecule at adherens junctions [19]. This cleavage occurs through an ATP-dependent mechanism and rapidly disrupts epithelial barrier integrity [19]. In HT29/C1 cells, the extracellular domain of E-cadherin is shed, followed by γ-secretase-mediated cleavage of the remaining membrane-tethered fragment, producing a 28 kDa cytoplasmic segment that is subsequently degraded via the proteasome [68]. This progressive disassembly of cell–cell contacts facilitate paracellular flux of ions and solutes, contributing to diarrhea.

Simultaneously, BFT exerts broad proteolytic activity against cytoskeletal and extracellular matrix components, including type IV collagen, actin, tropomyosin, myosin, fibrinogen, gelatin, and immune-related proteins such as complement C3 and α1-proteinase inhibitor. Disruption of the actin cytoskeleton induces characteristic morphological changes such as cell rounding and swelling, impairs structural integrity, and weakens epithelial resistance [38,45,69,70].

These structural alterations are closely linked to inflammatory signaling. BFT exposure activates MAPK and NF-κB pathways, promoting the secretion of proinflammatory cytokines like IL-8 and TNF-α [71]. Concurrently, it induces COX-2 and prostaglandin E2 expression, exacerbating fluid secretion and immune cell recruitment—key contributors to mucosal inflammation and secretory diarrhea [72].

Beyond acute epithelial injury, BFT drives tumorigenic processes. Cleavage of E-cadherin leads to β-catenin release, which activates oncogenic transcription, particularly in the context of adenomatous polyposis coli (APC) mutation, upregulating c-Myc and fostering hyperproliferation [30,73,74]. The toxin also upregulates anti-apoptotic factors such as cIAP2 and induces spermine oxidase (SMO), increasing reactive oxygen species (ROS), DNA damage, and compensatory epithelial proliferation. Genotoxicity is further evidenced by the activation of H2AX, a marker of DNA double-strand breaks [75,76].

In APC^Min/+^ mouse models, colonization with enterotoxigenic *B. fragilis* (ETBF) induces STAT3 activation and a robust Th17 cytokine profile (IL-17, IL-23, IL-6, TGF-β), generating a pro-carcinogenic inflammatory milieu [77]. Moreover, BFT has been linked to the activation of a STAT3/ZEB2 signaling axis, further compromising epithelial barrier integrity and promoting malignant transformation in CRC cells [25].

In parallel, epigenetic modulation also plays a role in ETBF-driven carcinogenesis. Recent findings indicate that ETBF can promote colorectal cancer cell proliferation through the downregulation of miR-149-3p, mediated by m^6^A RNA methylation via METTL14. This pathway affects the alternative splicing regulator PHF5A and its downstream target KAT2A. Since miR-149-3p also regulates Th17 cell differentiation, its progressive reduction in patients with IBD and CRC—inversely correlated with ETBF prevalence—underscores the ETBF/miR-149-3p axis as a potential therapeutic target for both inflammatory and neoplastic diseases of the colon [9].

## 4. Challenges in the Detection of *Bacteroides fragilis* and Future Research Directions

*Bacteroides fragilis* is a Gram-negative obligate anaerobic bacterium that resides in the human gastrointestinal tract [78]. Although it plays important roles in maintaining gut homeostasis, certain strains, ETBF, are implicated in inflammatory bowel diseases (IBDs) and CRC [54,78]. Accurate detection and identification of ETBF and its toxin, BFT, remain challenging due to multiple technical and biological factors.

The isolation and detection of *Bacteroides fragilis* from clinical samples require specialized culture media and strict anaerobic conditions, which complicate and prolong the cultivation process. Furthermore, the high microbial competition present in complex specimens such as feces and colorectal mucosa, along with the relatively low abundance of ETBF during asymptomatic colonization, significantly complicates its isolation and detection. Although selective media such as Bacteroides bile esculin (BBE) agar are commonly employed under anaerobic conditions, overgrowth of other anaerobic organisms, such as enterococci and lactobacilli, can interfere with the isolation of B. fragilis [79].

Traditional biochemical identification methods, such as Rapid ID32A and API 20A kits, are limited by low specificity and often fail to distinguish B. fragilis from closely related species. However, it has been reported that this biochemical scheme is not as accurate as a multiplex PCR identification scheme [80].

While advanced technologies like matrix-assisted laser desorption–ionization time-of-flight mass spectrometry (MALDI-TOF MS) offer higher specificity by analyzing ribosomal protein profiles [81,82], their high cost and technical requirements limit their availability in resource-constrained settings. The Sakamoto study demonstrated that MLSA based on six housekeeping genes (dnaJ, gyrB, hsp60, recA, rpoB, and 16S rRNA) is a valuable method for the identification and classification of Bacteroides species [83]. However, although this method is highly accurate, the need for PCR and sequencing limits its widespread use in all diagnostic laboratories.

Additionally, the confirmation of toxin production by *Bacteroides fragilis* strains has traditionally relied on biological assays such as the ileal loop test [56,59]. However, this method is costly, time-consuming, and not feasible for routine use in many laboratories.

Alternatively, cell-based diagnostic assays utilizing HT29/C1 cell cultures have been developed and were historically used for BFT detection due to their ability to detect less than 0.5 picomoles of toxin with high specificity and sensitivity [84]. However, this method is now considered outdated due to its labor-intensive procedures, and the availability of more advanced and standardized technologies. Thus, while informative in earlier research, it is no longer regarded as a practical tool for routine clinical diagnostics.

The most common method for identifying *Bacteroides fragilis*, the *bft* gene, and its different isotypes is PCR. The 16srRNA gene, nanH, gyrB, and leuB are widely used for the identification of different Bacteroides species [85,86,87]. However, studies have demonstrated that the accuracy of detecting toxigenic strains can be significantly influenced by the design of PCR primers. Some research findings indicate that certain commonly used primers may preferentially detect only specific isotypes of the *bft* gene, such as *bft-1*, while exhibiting reduced sensitivity toward *bft-2* and *bft-3*. These observations underscore the importance of designing primers with comprehensive coverage of all bft isotypes to prevent inaccurate estimations of ETBF prevalence in clinical and epidemiological investigations [88]. Moreover, in addition to the proper design of primers for *bft* detection, the choice of PCR methodology plays a critical role in the sensitivity and accuracy of diagnosing enterotoxigenic ETBF. Comparative studies have demonstrated that, when using purified bacterial DNA, different PCR approaches, including SYBR Green qPCR, TaqMan qPCR, and digital PCR (dPCR), exhibit comparable sensitivity for detecting the toxin gene. However, when applied to clinical stool samples, substantial differences in sensitivity were observed, with SYBR Green qPCR detecting significantly fewer positive cases compared with TaqMan qPCR and dPCR. While TaqMan and dPCR methods identified the bft gene in over 90% of samples, SYBR Green qPCR detected positivity in only about one third of cases [89].

In addition, the type of clinical sample significantly affects detection rates. Biopsy specimens of colorectal mucosa consistently show higher *bft* positivity than fecal samples [63]; however, ethical considerations, cost, and invasiveness limit their routine clinical application. Other confounding factors include variability in microbial load, host immune status, and geographic differences in ETBF prevalence, indicating that detection strategies must be adapted to population-specific contexts. Furthermore, many previous studies suffer from limitations such as small sample sizes and methodological heterogeneity, including variations in sample collection, DNA extraction, and PCR protocols, as well as reliance on *bft* gene detection without assessing actual toxin expression or biological activity. Notably, one of the most critical challenges involves the accurate assessment of BFT’s biological activity. While several methods can detect the presence of the *bft* gene or its protein product, confirming that the active toxin is being produced and is functionally relevant remains technically difficult, particularly in complex clinical samples such as feces or tissue biopsies. These limitations are consistent with findings from the systematic review by Scott et al. (2022), which emphasized the high methodological heterogeneity, poor reproducibility, and risk of bias in many in vitro and in vivo studies assessing ETBF detection and its role in CRC [31]. The lack of standardized protocols and inconsistent reporting across studies continues to hinder the development of reliable diagnostic strategies.

These gaps highlight the need for multicenter studies using standardized protocols and incorporating multi-omics approaches (genomics, transcriptomics, proteomics).

Recent advancements offer promising new directions. For example, monoclonal antibodies and nanobody-based assays have been developed for the sensitive and specific detection of BFT1 and BFT2, enabling the creation of ELISA platforms with detection limits as low as 20–40 ng/mL [90]. Additionally, high affinity nanobodies targeting catalytic and pro-domain regions of BFT provide opportunities for both diagnostic and therapeutic applications [91]. Moreover, recent studies have identified approved drugs capable of inhibiting BFT-3 by binding to an allosteric site within its proenzyme structure, thereby blocking toxin maturation and activity [92]. Moreover, studies have shown that some probiotic *Bifidobacterium longum* BB536 may be effective in reducing the number of ETBF and other intestinal pathogenic bacteria by improving mucosal barrier function [93,94]. Therefore, investigating the role of non-toxigenic *Bacteroides fragilis* (NTBF) strains as potential probiotics in reducing ETBF colonization is a topic of considerable interest for future research.

Finally, several critical needs remain unmet. There is a need for multiplex diagnostic platforms capable of simultaneously assessing the presence of the *bft* gene, its transcript expression, and BFT protein activity. Standardized affordable diagnostic kits suitable for routine clinical use must also be developed. To better understand ETBF pathogenesis, large multicenter studies using standardized methods and integrated omics data remain urgently needed. Furthermore, research efforts should focus on developing targeted strategies for neutralizing BFT activity, including small molecule inhibitors, antibody-based therapies, and competitive inhibitors that can block toxin–receptor interactions. Such approaches may provide new therapeutic options for managing ETBF-associated diseases.

## 5. Conclusions

Considerable progress has been made in better understanding the role of ETBF and its toxin in gut-associated diseases, yet important challenges remain. While the contribution of BFT to epithelial barrier disruption and inflammation is well recognized, the precise molecular mechanisms—especially the direct interaction with host targets like E-cadherin—require further investigation.

Current diagnostic approaches are often limited by suboptimal sensitivity, specificity, and an inability to confirm active toxin production. Promising strategies, including nanobody-based detection methods, multiplex assays, and targeted therapeutics are being explored to overcome these limitations. The use of probiotics that enhance epithelial barrier function and regulate immune responses offers a complementary non-invasive approach to controlling ETBF colonization. Moving forward, integrative efforts combining advanced diagnostics, toxin-neutralizing strategies, and multi-omics research will be critical to improve clinical management and develop targeted therapies for ETBF-associated diseases.

## Data Availability

No new data were created or analyzed in this study.

## Abbreviations

AAD	Antibiotic-associated diarrhea
BfPAI	*Bacteroides fragilis* pathogenicity island
BFT	*Bacteroides fragilis* toxin
CRC	Colorectal cancer
CDI	Clostridioides difficile infection
CTn86	Conjugative transposon 86
DSS	Dextran sulfate sodium
ETBF	Enterotoxigenic *Bacteroides fragilis*
GI	Gastrointestinal
IL-10	Interleukin-10
IL-22	Interleukin-22
MUC-2	Mucin 2
NTBF	Non-toxigenic *Bacteroides fragilis*
OMVs	Outer membrane vesicles
PSA	Polysaccharide A
RprXY	Two-component regulatory system RprXY
STAT3	Signal transducer and activator of transcription 3
Treg	Regulatory T cell
ZEB2	Zinc finger E-box binding homeobox 2
ZO-1	Zonula occludens-1
